# Supplementary material for: Chromothripsis during telomere crisis is independent of NHEJ, and consistent with a replicative origin
Source: Genome Res. 2019 May;29(5):737–49. doi: 10.1101/gr.240705.118 (PMC6499312; doi:10.1101/gr.240705.118)
Supplement: Supplemental Material [file supp_gr.240705.118_Supplemental_file_1.zip › contigs/annotated_contigs/DB55/contig.2.DB55_length_481_mean_cov_8.13513513514.docx]

**DB55_length_481_mean_cov_8.13513513514**

```
GGTCCCCTGGAGGTGGGACCCTCCTGGGTGACTCTGGGCACTGCCATCCAACCCCACATGCCCCACCTGGCTGCTTCGCTCACACCCCC
 >chr9:133862263-133862570 - E=3e-173
CAGTCTCCCACTTCCACGCACAAGAATCTTTCCTTTCCTTTGGCTACAAACTGGGGGAGGGGAGGACGACCCAATTTATTTGGCAACAA

ATAAAGAGCTGCCATGATTAAGATGGGGGGGAAGTTTAAAGGAGGAAAAATACCTTGGGTAATGCCATCTCCTGCGTCTTTATATTCTG

AAACGGATCGTTCTGGACATGGGCAGAAGGCCTGATTTAA|GTGTAGCCA|GGATTACAGGCGTGAGCCACAACACCCAGCTAATTTTT
                                                  >chr2:202862964-202863129 - E=9e-41
GTATTTTTAGTAGAAACGGGGTTTCACCATGTTGACCAGGCTGGTCTCGAACTCCTGACCTCAGGTGATCACCTGCCTCAGCCTCCCTG

AGTGCTGGGCTTACGGACATGAGCCACCACGCCCAGC|C
```
